# Sex-biased intron retention in Alzheimer’s disease

**DOI:** 10.4103/NRR.NRR-D-25-00456

**Published:** 2025-09-03

**Authors:** Ching-Thong Choo, Chin-Tong Ong

**Affiliations:** Temasek Life Sciences Laboratory, Singapore; Department of Biological Sciences, National University of Singapore, Singapore

**Higher prevalence of sporadic Alzheimer’s disease in women:** Alzheimer’s disease (AD) is a progressive neurodegenerative disorder caused by the accumulation of amyloid-β (Aβ) plaques and Tau neurofibrillary tangles in the affected brain regions. The clearance of these pathological protein aggregates by microglia can trigger excessive neuroinflammation, which contributes to brain atrophy. AD exhibits clinical heterogeneity and is characterized by highly complex, multifactorial etiology (Lopez-Lee et al., 2024). In the rare autosomal dominant forms of familial AD, patients inherit mutations in genes encoding amyloid precursor protein or presenilins. In contrast, the majority of the cases are classified as late-onset sporadic AD, in which aging is the primary risk factor. Disease susceptibility is sex-biased, with approximately 1 in 5 women at risk of developing AD during their lifetime, compared to 1 in 10 men. Beyond socio-cultural factors, a previous study suggests that inherent biological differences between the sexes contribute to the higher prevalence of AD in women (Lopez-Lee et al., 2024). For example, hormonal fluctuations during menopause can disrupt metabolic and inflammatory processes, increasing vulnerability to AD. Sex-specific differences in gene regulation and autophagy may also significantly impair protein homeostasis in females, thereby exacerbating AD pathology. Identifying other sex-biased mechanisms will deepen our understanding of AD etiology and lead to more effective treatment.

**Increased intron retention is linked to sporadic AD:** Multimodal analyses of the dorsolateral prefrontal cortex (DLPFC) from large AD cohorts have revealed significant discrepancies between transcriptional and proteomic changes associated with AD progression (Johnson et al., 2022). These findings underscore the proteopathic nature of AD, suggesting that disruption in protein homeostasis, independent of RNA level, may contribute to the rate of cognitive decline. Protein abundance is likely influenced by other mechanisms such as RNA splicing, post-translational modifications, and regulation of translation and degradation. Increased intron retention (IR), an alternative form of RNA splicing, has been shown to be associated with AD brains (Adusumalli et al., 2019). Combined-sex analysis of larger ROSMAP and Banner cohorts further revealed a higher number of differential intron retention (DIR) events in the DLPFC of AD patients compared to healthy controls (Choo et al., 2025). Notably, more than 90% of these computationally defined retained introns were experimentally validated by reverse transcription polymerase chain reaction (Adusumalli et al., 2019; Choo et al., 2025). The AD cases were further stratified according to Braak stage (a measure of Tau neurofibrillary tangle pathology), CERAD score (Consortium to Establish a Registry for AD; a neuropathological assessment of Aβ plaques burden), and cogdx value (a clinical diagnosis of cognitive status). Importantly, a strong positive correlation was observed between IR levels and AD severity, as defined by increased pathological Tau/Αβ loads and cognitive decline, suggesting that DIR at specific genes may contribute to AD pathology and cognitive impairment (Choo et al., 2025).

**Elevated intron retention only in female AD DLPFC:** To investigate the effects of sex on DIR patterns and their possible consequences in AD progression, we performed transcriptomic analysis separately on the male and female AD cohorts. In female AD DLPFC, 1645 elevated DIR events were observed compared to healthy controls, while 233 DIR events were decreased. In stark contrast, the male cohort exhibited significantly fewer changes, with only 80 elevated DIR events in AD and 125 in controls (Choo et al., 2025). Notably, very few DIR events were detected between healthy male and female individuals (*n* = 29; *P* < 0.05), suggesting that sex-specific DIR changes are largely AD-associated. Differentially retained introns exhibit higher G/C content and are shorter in length compared to fully spliced introns. Moreover, 233 genes with increased DIR in the female AD DLPFC overlapped with curated AD-associated genes from DisGeNET database and were linked to molecular functions such as ubiquitin-like protein ligase and Tau binding (Choo et al., 2025). Since many of these functions are dysregulated during disease progression, these findings suggest that DIR may contribute to female-specific AD pathology.

**Sex-specific transcriptomes and proteomes in AD DLPFC:** Sex differences in gene and protein expression have been well-documented during brain development and across various neurodegenerative diseases (Lopez-Lee et al., 2024). To explore this in the context of AD, we first identified pathways perturbed at both the RNA and protein levels in a sex-specific manner. An integrative approach was then used to investigate how AD-related transcriptomic and proteomic changes within DLPFC may be influenced by DIR.

**Figure 1:**
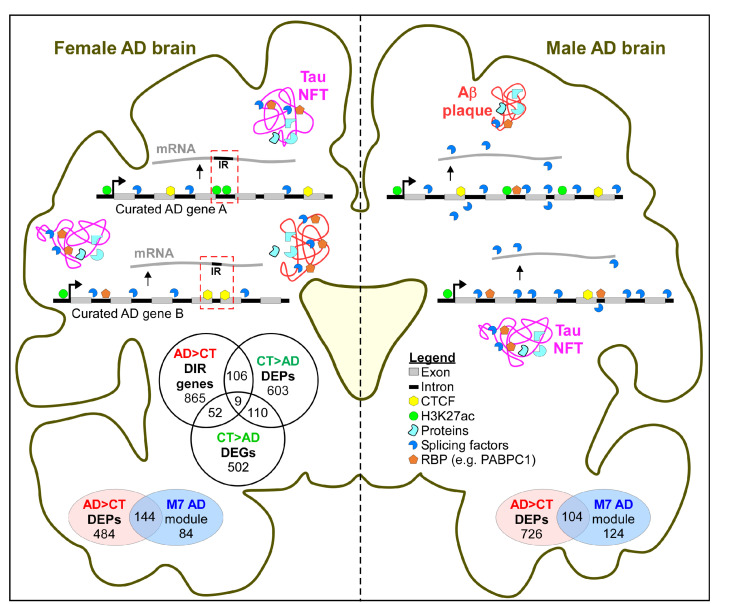
Mechanisms underlying sex-specific IR and protein expression patterns in AD brains. Increased IR in female AD brains correlates with differential binding of histone modification H3K27ac and the chromatin organizer CTCF at these retained intron sites (highlighted by red dotted box), compared to spliced introns. Difference in RNA splicing efficiency can also be influenced by the sequestration of RBPs by Aβ plaques or Tau NFT aggregates in females, or the elevated levels of RNA splicing factors in male AD brains. The significant overlap of genes exhibiting DIR with DEGs and DEPs suggests that IR may contribute to the changes in RNA and protein levels specifically in female AD brains. Furthermore, there was greater overlap between female DEPs with M7 mitogen-activated protein kinase/metabolism AD protein module compared to male DEPs in AD brains. Adapted from Choo et al. (2025). Created with CANVAS® (version 12). AD: Alzheimer’s disease; Aβ: amyloid-beta; CTCF: CCCTC-binding factor; DEG: differential expressed gene; DEP: differential expressed protein; DIR: differential intron retention; H3K27ac: histone H3 lysine 27 acetylation; IR: intron retention; NFT: neurofibrillary tangles; PABPC1: poly(A)-binding protein cytoplasmic 1; RBP: RNA-binding protein.

Distinct sex-specific molecular signatures were observed among AD-related differentially expressed genes and differentially expressed proteins (DEPs) in the DLPFC. For example, DEPs upregulated in male AD DLPFC were enriched for functions related to RNA splicing, localization, and transport (Choo et al., 2025). This suggests the activation of protective mechanisms in male AD brains that help maintain proper RNA splicing and processing. The absence or attenuation of such mechanisms may contribute to the increased aberrant IR observed in female AD patients. Compared to males, a higher percentage of upregulated DEPs in female AD brains overlapped with proteins in the mitogen-activated protein kinase/metabolism M7 module, which is strongly associated with the rate of cognitive decline (Johnson et al., 2022; **Figure 1**).

Integrative analysis revealed that IR levels were inversely correlated with mRNA expression. The reduced expression of mRNA transcripts with retained introns is indicative of nonsense-mediated mRNA decay, a cellular surveillance mechanism that identifies and degrades faulty mRNAs, often those containing premature termination codons. Interestingly, in female AD DLPFC, about 10% of genes with increased IR also showed reduced protein expression compared to controls, independent of changes in their mRNA levels (**Figure 1**). Downregulated DEPs encoded by these DIR genes were primarily involved in synaptic function and cellular transport processes — pathways frequently disrupted during AD progression. However, it is important to acknowledge the limitations of inferring causative roles for IR in RNA and protein changes based solely on correlative analyses within the DLPFC. This is because the downstream effects are influenced by the positions of retained intron within the gene body. For instance, IR at the 5’end may interfere with ribosome binding and translation initiation. In contrast, IR occurring within or near the 3’end of mRNAs may produce alternative protein isoforms with altered stability and biochemical properties. Biochemical studies, along with the generation of antibodies against novel isoforms translated from mRNAs containing retained introns, will provide unequivocal evidence for the functional roles of IR (Ngian et al., 2022). Retained introns can be used to generate a new mass spectral reference library of predicted amino acid sequences, enabling interrogation of mass spectrometry fragment data from AD cohort to validate the translation of novel protein isoforms derived from transcripts with aberrant IR. Analysis of additional brain regions will help further elucidate the broader roles of disease-related IR in AD pathology.

**Epigenetic regulation of differential IR in AD:** mRNA splicing occurs co-transcriptionally and is regulated by distinct chromatin signatures that influence the recruitment of RNA-binding proteins (RBPs) and spliceosome components (Petrova et al., 2022). It is plausible that sex-specific epigenetic perturbations contribute to the distinct DIR patterns observed between male and female AD DLPFC. AD-specific enrichment of H3K27ac histone mark has been observed at genes associated with chromatin regulation, transcription, and disease-related pathways, suggesting a dysregulated feedback loop involving epigenetic states and gene activities during AD progression (Nativio et al., 2020). Our integrative analysis revealed significantly elevated levels of H3K27ac mark at the upstream and intronic regions of differentially retained introns, compared to fully spliced introns, in the female AD DLPFC (Choo et al., 2025). In primary human myeloid and lymphoid cells, increased chromatin accessibility has been shown to contribute to cell-type specific IR patterns (Petrova et al., 2022). Although H3K27ac is an active mark associated with open chromatin, further experiments are needed to elucidate how its differential binding regulates IR in female AD brains.

The nuclear zinc finger protein CTCF, which plays a crucial role in genome organization, can regulate alternative splicing, such as exon inclusion, by modulating RNA polymerase II pausing and chromatin looping (Alharbi et al., 2021). In female AD brains, reduced CTCF binding at genes involved in synaptic organization correlates with their decreased mRNA expression compared to healthy controls (Patel et al., 2023). Unlike the spliced introns, we observed elevated levels of CTCF binding at the 5′ and 3′ regions flanking the differentially retained introns in female AD brains. It is plausible that CTCF regulates IR either by impeding RNA polymerase II elongation, thereby impairing spliceosome recruitment, or through chromatin looping mechanisms. Similar CTCF binding patterns were observed at these differentially retained introns in both healthy and AD DLPFC, suggesting that specific post-translational modifications or alterations in its interacting partners, such as the cohesin complex, may precede CTCF-mediated regulation of IR in AD brains.

Pre-mRNA splicing is mediated by nuclear speckles, non-membranous structures composed of RNAs, RBPs and spliceosome components (Lester et al., 2021). RBP motif analysis revealed enrichment of KHDRBS3, SART3, and PABPC1 at differentially retained introns, implicating them in aberrant IR in female AD brains (Choo et al., 2025). Notably, pathological Tau aggregates have been shown to disrupt the composition and dynamics of nuclear speckles in both AD brains and mouse models (Lester et al., 2021). Mislocalization of RBPs and splicing machinery components can reduce their availability to nascent transcripts, leading to impaired splicing efficiency. For instance, aberrant retention of intron 11 in *Tau* gene results in the translation of truncated Tau11i species, which forms Sarkosyl-insoluble aggregates, suggesting that DIR may directly contribute Tau aggregation (Ngian et al., 2022). Compared to males, the lower basal autophagy levels in females suggest that they may be more susceptible to protein aggregation. Collectively, these findings suggest that DIR in female AD brains may be driven by a combination of sex-specific epigenetic signatures and the sequestration of RBPs or splicing machinery by Tau/Aβ aggregates (**Figure 1**).

**Predicted DIR in specific neuronal subtypes:** A single-cell atlas of the prefrontal cortex from 427 individuals with varying degrees of AD pathology revealed transcriptional signatures and specific neuronal subtypes associated with cognitive resilience and dementia (Mathys et al., 2023). The study also demonstrated a depletion of selectively vulnerable somatostatin inhibitory neuron subtypes in AD, whereas two distinct groups of inhibitory neurons were enriched in aged, cognitive resilient individuals. Leveraging the strong concordance between bulk and single-cell RNA-sequencing data, we investigated the neuronal subtypes in which DIR may occur in female AD brains. Due to low intronic read coverage in single-cell datasets, which limits accurate IR detection, we instead identified neuronal subtypes exhibiting significant downregulation of DIR genes based on the established negative correlation between IR and gene expression levels. Statistically significant downregulation of the DIR genes was observed in specific inhibitory (GPC5 RIT2) and excitatory (RELN CHD7) neuronal subtypes from female AD patients. These transcriptional changes were absent in male counterparts and not observed in randomly selected genes.

Interestingly, the study also reported coordinated upregulation of the cohesin complex components in excitatory neurons and oligodendrocytes in association with global AD pathology (Mathys et al., 2023). Given that the cohesin complex is essential for CTCF-mediated chromatin looping, these findings underscore the importance of investigating cohesin-CTCF crosstalk in the regulation of DIR in AD. Future experiments could include genome-wide profiling of cohesin components as well as mapping 3D chromatin structures around differentially retained introns by chromosome conformation capture (3C)-based techniques in AD brain tissues.

**Concluding remarks:** Our findings suggest that sex-specific increase in aberrant IR at numerous curated AD-associated genes may be linked to the higher prevalence of AD in women. However, it is important to recognize that IR also plays key regulatory roles during tissue development and homeostasis. These include the nuclear sequestration of long transcripts, allowing for their rapid cytoplasmic export in response to neuronal activity, as well as expansion of the proteomic repertoire. Indeed, a novel non-aggregative Tau isoform containing retained intron 12 was found to be reduced in AD patients compared to healthy controls, suggesting a potential protective role against Tau pathology (García-Escudero et al., 2021). Elucidating the roles of genes exhibiting increased IR in healthy controls compared to AD brains (125 in males and 233 in females) may offer new insights into how alternate splicing events regulate AD pathology and inform the development of more effective therapeutic strategies.

*This work was supported by Temasek Life Sciences Laboratory core funding (3160) (to CTO).*
